# Impact of chronic endometritis treatment in patients with reproductive failure

**DOI:** 10.5935/1518-0557.20260049

**Published:** 2026

**Authors:** Sara Vázquez Ramos, Alejandro Olloqui Escalona, Jorge García Ibáñez, Laura de la Fuente Bitaine, Carmen Guillén Gámez, Concepción Pérez Sagaseta, Cristina González Macho, María Concepción Carrera Roig

**Affiliations:** 1 Department of Obstetrics and Gynecology, Hospital Universitario 12 de Octubre, Universidad Complutense de Madrid, Madrid, Spain

**Keywords:** chronic endometritis, reproductive failure, recurrent implantation failure, recurrent pregnancy loss, antibiotic therapy

## Abstract

**Objective:**

To assess whether the diagnosis and antibiotic treatment of chronic endometritis improve reproductive outcomes in patients with recurrent pregnancy loss or recurrent implantation failure.

**Methods:**

This retrospective study included 67 women with recurrent pregnancy loss or recurrent implantation failure. Chronic endometritis was diagnosed in 30 patients (44.8%) based on hysteroscopic findings, CD138-positive histology, or a positive endometrial culture. All patients diagnosed with chronic endometritis received first-line antibiotic therapy, most commonly doxycycline 200 mg/day for 14 days.

**Results:**

Following antibiotic therapy, 17 patients (56.7%) achieved a clinical pregnancy and 12 (40%) achieved a live birth. Clinical pregnancy and live birth rates were similar in women with recurrent pregnancy loss (60% and 40%, respectively) and those with recurrent implantation failure (55% and 40%, respectively), with no statistically significant differences between patients with resolved and persistent chronic endometritis (*p*>0.05).

**Conclusion:**

In this study, antibiotic treatment for chronic endometritis was not associated with a statistically significant improvement in reproductive outcomes. The heterogeneity of diagnostic and therapeutic approaches highlights the need for standardized criteria and well-designed prospective studies to clarify its clinical relevance.

## INTRODUCTION

Chronic endometritis (CE) is characterized by persistent inflammation of the endometrial mucosa, and is often associated with polymicrobial infections, endometrial dysbiosis, or immunological alterations (Ticconi *et al.*, 2024). While many patients remain asymptomatic, CE has been increasingly associated with recurrent implantation failure (RIF) and recurrent pregnancy loss (RPL) (Cicinelli *et al.*, 2019; Ticconi *et al.*, 2024).

The reported prevalence varies widely (3%-68%) depending on the population studied and the diagnostic criteria applied (Cicinelli *et al.*, 2019; Li *et al*., 2021; Liu *et al.*, 2022; Ticconi *et al.*, 2024), highlighting the heterogeneity of diagnostic criteria and study populations.

Diagnosis is typically based on a combination of hysteroscopy, endometrial biopsy, and immunohistochemistry for CD138-positive plasma cells, with MUM1 recently proposed as an additional marker to improve diagnostic sensitivity (Santoro *et al.*, 2023). Several studies have suggested improved reproductive outcomes after antibiotic therapy, particularly when histological resolution is achieved (Cheng *et al.*, 2022; Liu *et al.*, 2022; Veiga *et al.*, 2023; Vitagliano *et al.*, 2022). However, variability in diagnostic approaches and treatment protocols still hinders the development of clear clinical guidance.

This study aimed to evaluate whether the diagnosis and treatment of CE improve reproductive outcomes in women with reproductive failure.

## MATERIAL AND METHODS

**Figure 1:**
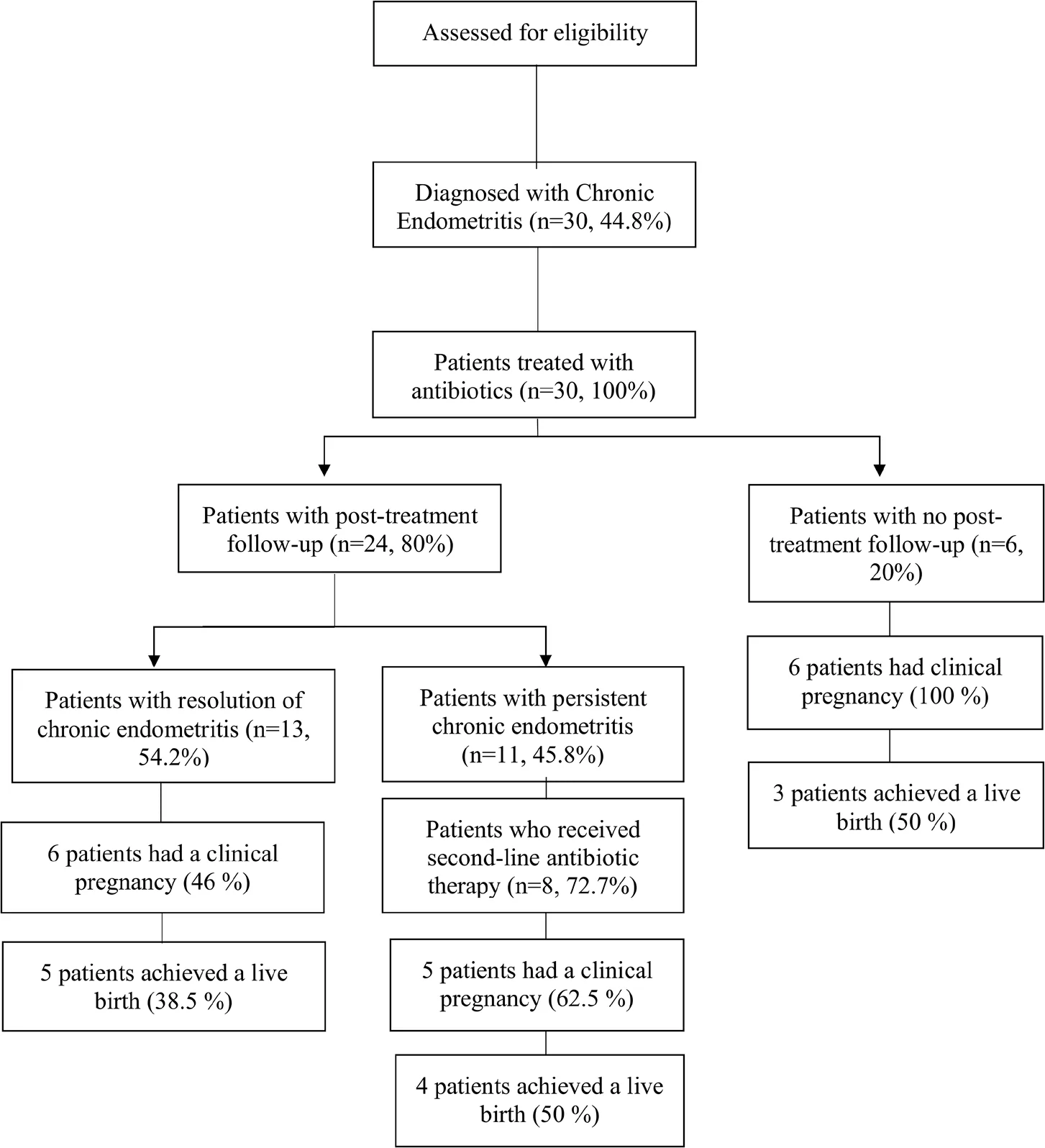
Treatment, post-treatment follow-up, and reproductive outcomes.

A retrospective study was conducted at a tertiary care hospital from January 2019 to December 2024, including 67 patients with a history of reproductive failure, as shown in Figure 1. Reproductive failure was defined as either RPL - two or more miscarriages according to the 2017 ESHRE Guideline (ESHRE Guideline Group on RPL *et al*., 2018) - or RIF - three or more unsuccessful embryo transfers with morphologically good-quality embryos, as established by the British Fertility Society (Mascarenhas *et al.*, 2022). Embryo quality was assessed according to the morphological classification described in the updated Istanbul Consensus Workshop on Oocyte and Embryo Morphological Assessment (Working Group on the update of the ESHRE/ALPHA Istanbul Consensus, 2025).

The inclusion criteria were women diagnosed with RPL or RIF who underwent hysteroscopic, histological, or microbiological evaluation for suspected CE. Exclusion criteria included loss to follow-up after diagnostic evaluation or refusal to receive antibiotic therapy.

All patients underwent diagnostic hysteroscopy with directed endometrial biopsy. In selected cases, microbiological cultures were obtained under hysteroscopic guidance. CE was diagnosed when at least one of the following criteria was met: (1) hysteroscopic features suggestive of CE, according to the criteria described by Cicinelli *et al*. (2019); (2) histological identification of at least one CD138-positive plasma cell per high-power field; or (3) positive endometrial culture.

All patients diagnosed with CE received first-line antibiotic therapy following Cicinelli *et al*. (2018) protocol, most commonly doxycycline 200 mg/day for 14 days. Post-treatment evaluation was performed by repeat biopsy or culture using a Cornier cannula. CE was considered resolved when no plasma cells or pathogens were detected. Otherwise, the condition was classified as persistent. Most patients with persistent CE received second-line antibiotic therapy, most commonly ceftriaxone 500 mg single dose with doxycycline 200 mg/day and metronidazole 500 mg/12 hours.

The primary outcome of this study was the live birth rate following CE treatment, defined as the proportion of patients who achieved at least one live birth after one or more embryo transfers (ESHRE Working Group on Recurrent Implantation Failure, 2023). The secondary outcome was the clinical pregnancy rate, defined as the proportion of embryo transfers resulting in pregnancy confirmed by an ultrasound-detected gestational sac (ESHRE Working Group on Recurrent Implantation Failure, 2023). Subgroup analyses compared outcomes between (1) women with RIF versus RPL and (2) patients with resolved versus persistent CE after treatment.

Continuous variables were tested for normality using the Shapiro-Wilk test. Qualitative variables are presented as absolute frequencies (n) and percentages (%), and quantitative variables as mean±standard deviation or median and interquartile range, as appropriate. The chi-square test, linear-by-linear association test, or Fisher’s exact test was used to assess associations between qualitative variables. Statistical analyses were performed using IBM SPSS Statistics 25.0, with *p*<0.05 considered statistically significant. The study protocol was reviewed and approved by the institutional ethics committee, which granted a waiver of informed consent because of the retrospective design and use of anonymized data. An anonymized dataset is available at Vázquez Ramos (2025).

## RESULTS

Among the 67 women with reproductive failure, CE was diagnosed in 30 (44.8%) based on one or more diagnostic criteria. Of these, 20 (66.7%) had RIF and 10 (33.3%) had RPL. The median age of the cohort was 35 years [32-36], with a mean anti-Müllerian hormone (AMH) level of 3.7±2.7 ng/mL and a median total motile sperm count (TMSC) of 7×10^6^ [1.5-25.6] (Table 1). Male factor infertility and ovarian factor infertility were each observed in 10 cases (33.3%) (Table 1).

**Table 1:** Clinical characteristics and infertility factors of patients with chronic endometritis

Clinical characteristics	Value (n=30)
Age (years), median (IQR)	35 [32-36]
AMH^*^ (ng/mL), mean±SD	3.7±2.7
TMSC† (×10^6^), median (IQR)	7 [1.5-25.6]
Infertility factors Male infertility factor, n (%) Ovarian factor, n (%) Endometriosis, n (%) Advanced age, n (%) Low ovarian reserve, n (%) Uterine factor, n (%) Tubal factor, n (%)	10 (33.3%) 10 (33.3%) 4 (13.3%) 4 (13.3%) 4 (13.3%) 3 (10.0%) 2 (6.7%)
^*^Anti-Müllerian Hormone† Total Motile Sperm Count	

Histological detection by CD138 immunostaining was the most common diagnostic approach (86.7%), followed by hysteroscopic findings (all showing endometrial micropolyps) (26.7%) and positive cultures (20%). The isolated microorganisms are shown in Table 2.

**Table 2:** Microorganisms isolated in cultures

Microorganisms	Value (n=30)
*Streptococcus anginosus*, n (%)	2 (33.3%)
*Mycoplasma hominis* / Ureaplasma, n (%)	2 (33.3%)
*Lactobacillus acidophilus*, n (%)	1 (16.7%)
*Enterococcus faecalis*, n (%)	1 (16.7%)

Most patients received doxycycline 200 mg/day for 14 days as first-line therapy (n=20; 66.7%). The remaining 10 patients received ciprofloxacin 500 mg every 12 hours, amoxicillin-clavulanic acid 875/125 mg every 8 hours, or combined antibiotic regimens, as detailed in Table 3. Post-treatment evaluation was performed in 24 patients (80%), confirming CE resolution in 13 (54.2%) and persistence in 11 (45.8%). Among those with persistent CE, 8 (72.7%) received second-line antibiotic therapy, most commonly ceftriaxone 500 mg as a single dose followed by doxycycline 200 mg/day and metronidazole 500 mg every 12 hours. In the remaining cases, treatment included cefixime 400 mg/day, amoxicillin-clavulanic acid 875/125 mg every 8 hours, or a combination of metronidazole 500 mg every 12 hours and ciprofloxacin 500 mg every 12 hours (Table 4).

**Table 3:** First-line antibiotic therapy

First-line antibiotic therapy	Value (n=30)
Doxycycline 200 mg/day, n (%)	20 (66.7%)
Ciprofloxacin 500 mg/12 hours, n (%)	3 (10%)
Doxycycline 200 mg/day + azithromycin 400 mg/day, n (%)	3 (10%)
Amoxicillin-clavulanic acid 875/125 mg/ 8 hours, n (%)	1 (3.3%)
Doxycycline 200 mg/day + ciprofloxacin 500 mg/12 hours, n (%)	1 (3.3%)

**Table 4:** Second-line antibiotic therapy

Second-line antibiotic therapy	Value (n=30)
Ceftriaxone 500 mg single dose + Doxycycline 200 mg/day + Metronidazole 500 mg/12 hours, n (%)	4 (36.4%)
Metronidazole 500 mg/12 hours + Ciprofloxacin 500 mg/12 hours, n (%)	2 (18.2%)
Cefixime 400 mg/day, n (%)	1 (9.1%)
Amoxicillin-clavulanic acid 875/125 mg/ 8 hours, n (%)	1 (9.1%)

Following antibiotic therapy, 17 patients (56.7%) achieved a clinical pregnancy and 12 (40%) achieved a live birth. On average, each patient underwent 1.37±0.85 embryo transfers after treatment. Among women with RIF, 55% achieved a clinical pregnancy and 40% achieved a live birth, whereas among those with RPL the rates were 60% and 40%, respectively (Table 5). Clinical pregnancy and live birth rates did not differ significantly between patients with resolved CE (46.2% and 38.5%) and those with persistent disease after second-line antibiotic therapy (62.5% and 50%) (*p*>0.05). However, the small sample size of this study limited its power to detect statistically significant differences. Nevertheless, the observed clinical pregnancy and live birth rates suggest a potentially favorable reproductive trend following antibiotic therapy.

**Table 5:** Reproductive outcomes according to type of reproductive failure

Type of Reproductive Failure	Number	Patients with a clinical pregnancy (n, %)	Patients with a live birth (n, %)
Recurrent implantation failure	20	11 (55 %)	8 (40%)
Recurrent pregnancy loss	10	6 (60%)	4 (40%)

## DISCUSSION

This study adds to the existing evidence on the role of CE in reproductive failure and the potential benefit of antibiotic therapy, showing a CE resolution rate of 54.2%, with 56.7% of patients achieving a clinical pregnancy and 40% achieving a live birth. Although no statistically significant differences were observed according to CE resolution status, these descriptive outcomes still suggest a potentially favorable reproductive trend after treatment.

Our results align with previous studies reporting variable CE resolution rates and reproductive outcomes following antibiotic therapy (Cicinelli *et al.*, 2021; Kitaya *et al.*, 2017; Sakai *et al.*, 2024; Veiga *et al.*, 2023; Vitagliano *et al.*, 2022; Xiong *et al.*, 2021). Despite the moderate resolution rate, the reproductive outcomes observed in our study were comparable to those reported in larger cohorts, supporting the clinical value of CE management. However, the heterogeneity of diagnostic methods, small sample size, and retrospective design limit the generalizability of our results.

The biological plausibility of CE’s negative impact on fertility is well-established. Persistent endometrial inflammation leads to leukocyte infiltration, cytokine and chemokine overexpression, and transcriptional dysregulation of immune and angiogenic pathways, together with impaired stromal decidualization, progesterone resistance, and abnormal uterine contractility (Di Pietro *et al.*, 2013; Kitaya & Yasuo, 2010; Pirtea *et al.*, 2021; Wu *et al.*, 2017). These mechanisms compromise endometrial receptivity and may explain the reproductive improvement observed after antibiotic therapy (Buzzaccarini *et al.*, 2020).

Potential confounders such as embryo quality, female age, and underlying infertility factors may also have influenced reproductive outcomes in our cohort. Nonetheless, all patients underwent hysteroscopy-guided biopsy, which ensured diagnostic accuracy and consistency, and most completed post-treatment follow-up, allowing a reliable evaluation of therapeutic response.

The main limitations of this study are its small sample size and retrospective design, both of which reduce statistical power and limit generalizability. Despite these constraints, the observed 40% live birth rate is consistent with previous reports and supports CE as a potentially modifiable condition affecting reproductive prognosis.

Additionally, while our study did not demonstrate significant differences based on post-treatment CE status, current evidence suggests that confirming histological resolution may improve reproductive outcomes (Cheng *et al.*, 2022; Liu *et al.*, 2022). Therefore, several authors advocate for the systematic post-treatment evaluations to optimize clinical management.

Future research should aim to investigate the role of CE as a modifiable risk factor for reproductive failure through large, prospective studies using standardized diagnostic and therapeutic criteria. It should also explore adjuvant strategies to enhance treatment efficacy. In this regard, Zou *et al*. (2023) reported improved implantation and pregnancy rates with combined antibiotic and corticosteroid treatment, opening new avenues for investigation.

## CONCLUSION

In this cohort, antibiotic treatment of CE was associated with clinical pregnancy and live birth rates of 56.7% and 40%, respectively. No statistically significant differences in reproductive outcomes were observed between patients with resolved and persistent CE after treatment. Given the small sample size and diagnostic heterogeneity, these findings should be interpreted cautiously; however, the observed trends are consistent with previous studies and support the clinical relevance of CE management in assisted reproduction. Larger prospective studies using standardized diagnostic and therapeutic criteria are needed to clarify its true impact on reproductive outcomes.
